# Leveraging the utility of pharmacogenomics in psychiatry through clinical decision support: a focus group study

**DOI:** 10.1186/s12991-019-0237-3

**Published:** 2019-08-08

**Authors:** Andrew Goodspeed, Nicolas Kostman, Trenton E. Kriete, Joel W. Longtine, Sean M. Smith, Peregrin Marshall, Wesley Williams, Cheryl Clark, Weston W. Blakeslee

**Affiliations:** 1 RxRevu, Inc, 2601 Blake Street Suite 450, Denver, CO 80205 USA; 2 The Mental Health Center of Denver, 4141 E Dickenson Pl, Denver, CO 80222 USA

**Keywords:** Focus group, Pharmacogenetics, Pharmacogenomics, Clinical decision support, Psychiatry, Mental and behavioral health, Electronic health record

## Abstract

**Background:**

Pharmacogenomics is starting to build momentum in clinical utility, perhaps the most in mental and behavioral healthcare. However, efficient delivery of this information to the point of prescribing remains a significant challenge. Clinical decision support has an opportunity to address this void by integrating pharmacogenomics into the clinician workflow.

**Methods:**

To address the specific needs of mental health clinicians at the point of care, we conducted 3 focus groups with a total of 16 mental health clinicians. Each 1-h focus group was designed to identify the desired clinical decision support features, with a particular interest in pharmacogenomics, and potential negative or unintended consequences of clinical decision support integration at the point of care in a mental healthcare setting. We implemented an iterative design to expand upon knowledge generated in prior focus groups. The results from the guided discussion in the first focus group were used to develop a mental health clinical decision support prototype. This prototype was then presented during the next two focus groups to drive the discussion.

**Results:**

This study has identified main themes related to the desired clinical decision support features of mental health clinicians, the use of pharmacogenomics in practice, and unintended and negative consequences of clinical decision support integration at the point of care. Clinicians desire a more complete picture of the medication history of patients and guidance to choose medications in relation to cost, insurance coverage, and pharmacogenetics interactions. Mental health clinicians agreed that pharmacogenetics is useful and impacts their prescribing decisions when the data are available. Several negative consequences of clinical decision support integration were identified including alert fatigue and frustration using the tool. Several points of contention were related to the integration of the clinical decision support with the electronic health record, including bidirectional flow of information, speed, location within workflow, and potential incompleteness of information.

**Conclusions:**

We have identified general and unique considerations of mental health clinicians with regard to clinical decision support. Clinical decision support that incorporates desired features while avoiding negative and unintended consequences will increase clinician usage and will have the potential to improve the care of patients.

## Background

One in six adults in the United States is living with some form of mental illness.^1^ Despite numerous advances in pharmacological interventions and treatment strategies, many patients with mental health or behavioral health issues remain compromised after treatment. This is primarily due to a combination of treatment ineffectiveness and side effects from available pharmacotherapies. For instance, the response rate to first-line treatment is only 50–60% [1] for major depressive disorder, which is projected to be the second most common disease by 2020 [2]. Meanwhile, many of these patients also have to deal with side effects [3]. Other illnesses like attention-deficit/hyperactivity disorder (ADHD) and schizophrenia have effective therapies, but they too are associated with a variety of adverse effects, which can reduce adherence to medications [4–7]. Collectively, the management of mental health represents an area still in need of advanced therapies and treatment strategies.

Drug metabolism, efficacy, and side effects can be driven by single nucleotide polymorphisms (SNPs) in many genes. Pharmacogenetic (PGx) biomarkers have emerged as a promising tool to predict the clinical efficacy of many prescribed medications, including antidepressants and antipsychotics [8–11]. Peer-reviewed studies have shown that the use of PGx in the clinic increases the efficacy of antidepressants and reduces healthcare costs by 67% [10–13]. However, prescribers should use caution, as there are still outstanding questions that prompted a recent warning from the Food and Drug Administration (FDA). This warning states that “the FDA is aware of genetic tests that claim results can be used to help physicians identify which antidepressant medication would have increased effectiveness or side effects compared to other antidepressant medications. However, the relationship between DNA variations and the effectiveness of antidepressant medication has never been established.”^2^

While the use of PGx has great promise, there are several challenges and barriers to address before widespread adoption in clinics. Clinicians often lack necessary information and education to interpret PGx results and implement those findings into prescribing decisions. The delivery format of PGx information (often a 20 + page pdf) also makes it difficult for clinicians to easily incorporate PGx data points into their clinical workflow. In addition, test result turnaround times, the lack of standardized result formats, cost, and reimbursement considerations also hinder the adoption of PGx in the clinic [14]. With more and more promising studies highlighting the potential impact of PGx on mental health prescribing, efficient delivery and comprehension of this information are critical. The integration of PGx into electronic health records (EHRs) through clinical decision support (CDS) will reduce many of these barriers and increase the inclusion of PGx to inform prescribing decisions at the point of care.

CDS is the process of providing clinical knowledge and patient information to enhance provider decisions and actions [15]. Computerized CDS improves prescribing decisions and reduces the patient’s time spent in hospitals [16]. Dozens of CDS tools have begun to incorporate PGx information, most for the benefit of cancer treatment [17]. To increase the effectiveness of CDS with PGx integration, several suggestions include: reducing the number of alerts to address provider alert fatigue, including relevant resources to interpret results, and integrating PGx into the clinicians’ workflow [18]. However, these are general recommendations, and some clinicians may have additional desires and requirements that are specific to their clinical specialty.

In the field of psychiatry, there is significant interest in improving patient outcomes through the use of PGx [10, 11, 19] and CDS [20, 21], but so far these initiatives have rarely been integrated together. We hypothesize that successful and comprehensive integration of CDS and PGx within the clinician’s EHR workflow will ultimately improve prescribing decisions and patient outcomes in psychiatry. However, before this notion can be tested, the necessary CDS features to enhance prescribing decisions specific to psychiatry must be explored. Additionally, how PGx is used and viewed in the field of psychiatry in the context of CDS is undocumented. The focus of this manuscript is to evaluate input from mental health clinicians on EHR-integrated CDS, PGx, and the reaction of psychiatric clinicians to a CDS prototype. We: (1) identify desired features associated with a mental health-specific CDS with incorporated PGx data, (2) determine how mental health clinicians approach, implement, and use PGx information, and (3) identify potential negative and unintended consequences of a mental health-specific CDS with incorporated PGx. This study will assist in the design and development of future mental health-focused CDS incorporating PGx information to improve patient outcomes.

## Methods

### Study design

**Fig. 1:**
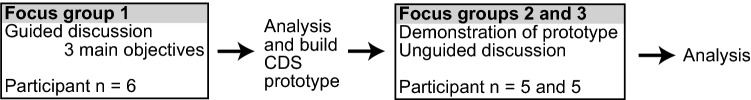
Schematic outline of this study. This study was comprised of three focus groups over two phases. The first phase used a focus group to obtain initial feedback on mental health-specific CDS and pharmacogenomics which was used to build a CDS prototype. The second phase consisted of two more focus groups, each beginning with a demonstration of the CDS prototype followed by an open discussion of CDS and pharmacogenomics in mental health. Finally, the data for all three focus groups were compiled and analyzed

We conducted this qualitative research study in two consecutive phases: (1) initial feedback to guide CDS prototype design and (2) demonstration and discussion of a CDS prototype (Fig. 1). Both phases incorporate focus groups with clinicians in the field of mental health. The first phase consisted of one focus group to determine CDS needs for clinicians and specific needs for those in mental health. These findings were analyzed to construct a mental health-specific CDS prototype. In the second phase, the CDS prototype was demonstrated to two focus groups followed by an open discussion. Our iterative study design was used to build upon knowledge throughout the study to best uncover the needs and optimal EHR workflow of mental health clinicians.

### Focus group participants

Focus group participants volunteered after monthly medical staff meetings. These clinicians consisted of 16 total nurse practitioners and physicians with board certifications in psychiatry and family medicine. The length of clinical practices for the participants ranged from less than 5 to over 15 years.

### Focus group discussion

Each of the three focus groups ran for 1 h each. The first focus group consisted of a guided discussion (see Table 1 for Focus Group 1 prompts). This guided discussion was aimed to identify desired CDS and PGx features, to determine how clinicians use PGx, and to identify potentially negative consequences of CDS integration, all in the field of mental health.

**Table 1:** Topic guide of focus group 1

Questions:
Could you describe your priorities when dealing with patients prior to prescribing medication?
How long do you have with each patient generally speaking?
What sorts of tools do you have to assist you with prescribing medications? Do they work well? Do you trust them?
How do you find past medication history?
What do you think about pharmacogenomics assays?
What pharmacogenomics information in the report is most useful?
What medical conditions are you more likely to use PGx for?
Sketch a CDS

Focus Groups 2 and 3 were conducted differently. First, the CDS prototype was demonstrated to the participants. The demonstration consisted of using a demo (proof of concept) software environment that walked focus group participants step-by-step through the features that were developed after the results of Focus Group 1 were analyzed. The demonstration was followed by an open discussion related to the CDS prototype with minimal prompting compared to the first focus group. When needed, we asked the participants for clarification of their discussion and prompted the participants with similar discussion points used in the first phase to continue the discussion.

### Focus group analysis

All focus groups were audio recorded, transcribed, and analyzed for themes. These themes were then categorized to identify major and recurrent themes from the participants. All identified themes were discussed by the co-authors. Quotes from study participants are included in the manuscript to supplement the presentation of each of the major findings.

### Development of the CDS prototype

The analysis from the first phase of the study was used to design and construct a mental health-specific CDS prototype that was later embedded into the EHR in the second phase of the study. Specific features to be included in the User Interface (UI) of the prototype were prioritized based on the data elements that were available in the EHR’s Consolidated Clinical Document Architecture (CCDA) payload. RxRevu subscribed to a CCDA feed from the Mental Health Center of Denver’s EHR, which alerted the platform whenever a patient record was opened, and provided the platform with a longitudinal record of labs, vitals, and medications. Using the EHR’s widgets (native software elements that could be used for CDS), the prototype would evaluate the patient’s current medications and check if their PGx assay was available. If present, an HTML view would be embedded in the EHR workflow showing if any gene–drug interactions were found on the patient’s current medication list, and what alternatives might be suitable, along with line charts showing the evolution of the patient’s clinical context.

## Results

### Participant characteristics and study design

Overall, 16 mental health clinicians participated in our 3 focus groups (Table 2). The participating clinicians had a wide range of ages and number of years practicing. The majority of the participants had a primary degree of MD/DO while the other three participants were nurse practitioners. All of the participants currently practice in an urban setting and see 25–50 patients per week.

**Table 2:** Focus group participant characteristics (*N* = 16)

Characteristic	Participants (%)
Age	
25–35.99	4 (25.0%)
36–45.99	4 (25.0%)
46–65.99	8 (50.0%)
Years of practice	
< 5	6 (37.5%)
5–9.99	3 (18.8%)
10–14.99	1 (6.3%)
Greater than 15	6 (37.5%)
Primary degree	
MD/DO	13 (81.3%)
NP	3 (18.8%)

Our study consisted of two phases (Fig. 1). Each phase consisted of focus groups with mental health clinicians lasting for 1 h each. In the first phase, a guided focus group was conducted covering desired CDS features, PGx in clinical practice, and potential negative and unintended consequences of CDS integration. In the conclusion of the first phase, a prototype mental health CDS platform was produced using the analysis from the first focus group. The second phase of the study consisted of two focus groups with an altered design from the first focus group. In this phase, the focus groups’ participants were shown a demonstration of the CDS prototype followed by an open discussion related to the prototype with minimal prompting. Collectively, the objectives of this study were to (1) identify the desired features associated with a mental health-specific CDS with PGx incorporation, (2) determine how mental health clinicians approach, implement, and use PGx information in clinical practice, and (3) identify potential negative or unintended consequences of a mental health-specific CDS with incorporated PGx.

### Development of a mental health CDS prototype

**Fig. 2:**
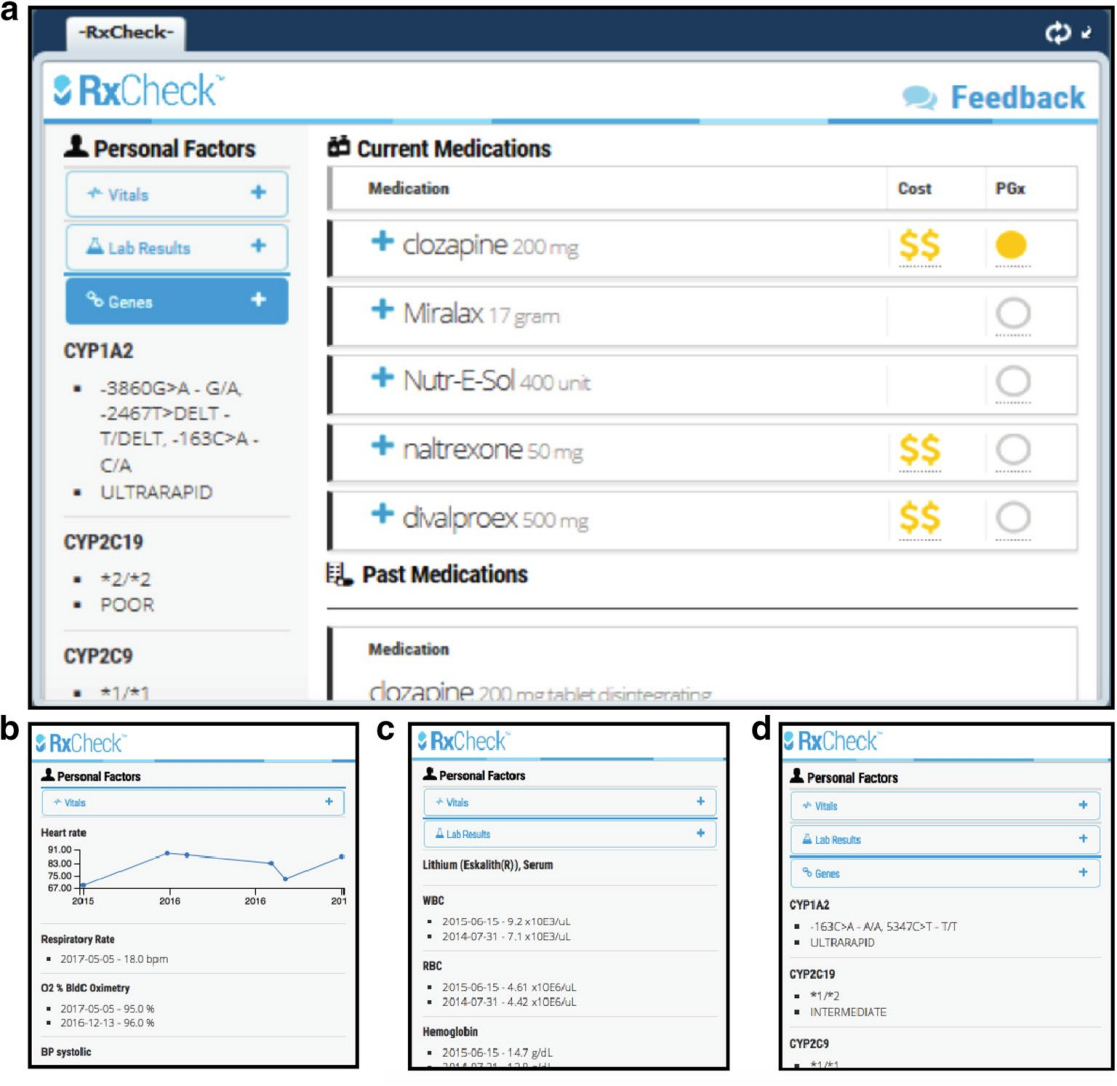
Screenshots of the prototype mental health CDS with pharmacogenomics integration. The CDS prototype consists of a main screen featuring tabs on the left and current and past medications on the right in the main panel (**a**). The tabs on the left can be expanded to show vitals (**b**), lab results (**c**), and pharmacogenomics information (**d**)

The results from the first focus group were analyzed to develop a prototype of a mental health-specific CDS containing many of the desired features identified from the first focus group. The CDS prototype was developed by the RxRevu software development team, as detailed in the methods. A screenshot of the prototype is provided in Fig. 2. The CDS prototype consisted of a primary dashboard displaying a patient’s: current medications, dosages, PGx guidance for current medications (if available), past medications, medication fill dates, and relative medication pricing (using $ signs based on average Medicare tier). On the left rail (the partitioned left side of the CDS prototype), dropdown drawers show additional information that can be displayed, including vital signs, lab results, and gene polymorphisms with pharmacologic implications (Fig. 2b–d). This prototype was demonstrated at the start of Focus Groups 2 and 3 to guide participant feedback. Patient identities were kept confidential throughout the duration of the study.

### Desired features and surfaced information of a mental health-specific CDS

**Fig. 3:**
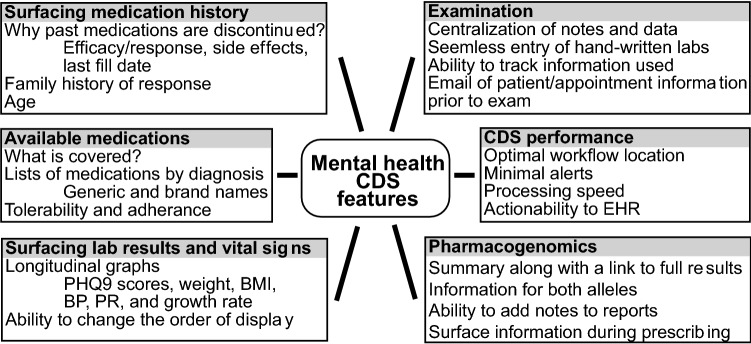
Desired CDS and pharmacogenomics features of mental health clinicians. The desired features of mental health clinicians are categorized and summarized

To construct an effective and widely used mental health CDS, it is necessary to determine the desired features of an appropriate CDS, the location of the CDS within the clinician workflow, and what information is useful to be surfaced by the CDS. The results from all three focus groups were compiled to provide the broadest feedback from all of the participating clinicians within this study. Throughout the study, mental health clinicians stressed the importance of surfacing previous medication history including prescription fill dates, as well as: lab results and vitals, available medications, examination information and notes, CDS performance, and features specific to PGx (Fig. 3).

#### Surfacing previous medication history

All of the participants agreed that past medication history is informative, important, and necessary to orient them toward appropriate treatment options for patients. The participants suggested notes for each past medication related to efficacy or response, side effects experienced, and last fill date would add much needed context to the past medication history: “*sometimes it is hard to figure out whether medicine was really tried or not…trying to figure out what has even happened to previous meds can be challenging*.” (Focus Group (FG) 1). Additionally, clinicians find the family history of response to medications to be important in choosing prescriptions for their patients. Finally, the age of the patient is important to clinicians at the point of care, particularly for children where clinicians tend to be more cautious with their prescribing decisions.

#### Listing available medications

Clinicians agreed that having a list of available medications (displayed in both brand and generic names) for each diagnosis within the CDS would be useful. Historical data on medication tolerability and adherence are also useful for clinicians at the point of prescribing. A feature identified by several mental health clinicians is the ability to know which medications are covered by the patient’s insurance plan: “*half the time I think of something and they are like that’s not going to be covered so I can’t use that*.” (FG1). Similarly, clinicians described interest in knowing the cost of medications to their patients. They describe how the cost can differ at pharmacies within the same region and that predicted cost information would assist them in their prescribing decisions.

#### Surfacing lab results and vital signs

Clinicians use many pieces of data to inform their decision at the point of care, and agree that presenting pertinent lab results and vital signs in the CDS would be an important feature. During examinations, the mental health clinicians who participated in this study prioritize longitudinal PHQ9 scores, weight, BMI, blood pressure, pulse rate, and growth rate. Some clinicians valued a display with all of the possible information displayed at once, while others were weary of too much information in one display: “*I want a one*-*stop shop where it all shows up on the same page and points me in that direction so I probably want more information*” (FG1) but that “*too much data doesn’t really solve the problem*” (FG1). Another feature identified relating to the presentation of vital signs and lab results is the ability of a clinician to change and customize the order of display based on their own preferences. Clinicians agreed they would participate in a one-time setup to customize their CDS display. There was a disagreement as to whether in-person training, videos, or a document would be the best method to provide customization instructions.

#### Examination notes

Prior to the examination, some clinicians would find value in an email displaying patient information to prepare for the appointment. The ability to track which displayed information was evaluated and used during the examination was an important feature to some clinicians. Seamless entry of hand-written lab reports is a feature that clinicians felt was lacking in their EHR, and would be useful in a CDS. Finally, an important CDS feature that came up at multiple times across the focus groups was centralization of notes and data: “*there is data in two different places and neither one has… like there’s no specific widget or place to go that says these are all the meds they were prescribed and this was their reaction to each one or what happened…and we inevitably probably miss things and try things again that were tried and had a good trial but we just couldn’t find it*.” (FG1).

#### CDS performance features

When prompted to describe where in the workflow CDS would be most useful, a clinician responded: “*when ordering meds*.” (FG1). Additionally, clinicians agreed that alert fatigue is important to avoid. Alert fatigue occurs when a clinician receives many unhelpful alerts from a system and reverts to ignoring most or all alerts [22]. Important alerts are necessary but unnecessary alerts negatively impact the clinician. One example of a requested alert was specific to Absolute Neutrophil Count (ANC) testing for patients taking clozapine: “*If it indicated how frequent you were needing ANC for patients with Clozaril*.” (FG3). The clinicians also describe several features that relate to the actionability of the CDS with the EHR. Clinicians desire the ability to order medications directly from the CDS and the ability to place information *into* the EHR using CDS. However, not all EHRs allow this level of actionability for third-party CDS. Finally, an unexpected CDS feature described by the participants related to computational performance and the necessity of the speed of the CDS: “*If its slow, if it takes 10* *s to load just a blank note for me to fill, then it will take a longer time for me to load the information to the app*.” (FG2).

#### Pharmacogenomics-related features

The common red/yellow/green standard of categorizing medications into groups based on predicted PGx efficacy appears to be well accepted [8–11]. All clinicians agree that they wish to see a color-coded summary of the PGx report, including information for genotypes of both alleles. Similar to other points of the discussion, clinicians desire a feature with the ability to add notes to the PGx report. Finally, clinicians wished for better integration of PGx information into their workflow with even the potential of orderable medications being colored by their interaction suggested by the PGx report: “*What I do wish I guess is that if part of the requisition, there was a list of meds that would be highlighted on top of the report so that I know to go where those meds are within it*.” (FG3).

### The use of PGx information by mental health providers

PGx has been shown to enhance personalized medicine and efficacy of care in the field of mental health [10–13]. One of the objectives of this study was to identify how mental health clinicians decipher, use, and trust PGx reports so that CDS can provide adequate features and information at the point of prescribing (Table 3).

**Table 3:** Main topics and findings discussed relating to pharmacogenomics and potential unintended negative consequences

Summary of major points addressed from the focus groups		
	Use of pharmacogenomics	Potential negative consequences
Major points raised during discussion	*The results summary is generally sufficient* Physicians do consult the full report if: Unfamiliar with vendor Desire in-depth analysis Clinical consideration *PGx is generally not used as a first step* Generally saved for poor responders or bizarre symptoms Can be used to satisfy worried parents and patients *Providers-PGx trust is important to act on PGx results* Providers are more likely to trust familiar vendors Allele and metabolizer information is important *Physical PGx reports may be handed to patients* To serve as a prop To allow patients to supply other providers with PGx results *Current shortcomings with using PGx at the point of care* Clinicians have difficulty incorporating PDF reports in their workflow PGx information does not integrate with patient information such as current and prospective medications at the point of prescribing	*Alert fatigue* Unnecessary alerts should be avoided to limit distraction from important alerts However, a lack of critical alerts is also negative *Difficulty to input relevant information* This type of information includes: Ratings/scores from third party vendors Hand-written lab reports *Addition of time to the length of visit* Providers generally only have 30 min for an examination Providers are unlikely to use tools that increase time Proper position of the CDS within the workflow will increase efficiency *Clinicians may lack trust in displayed data* Past experiences with CDS suggest not all relevant data are surfaced properly Confirmation of the accuracy of information will ease some of these concerns *Frustration using CDS* Clinicians often deal with slow and inconsistent CDS Clinicians will discard cumbersome CDS

#### Summary of PGx report is important to clinicians

Clinicians felt that, in most cases, the PGx report summary as provided by the PGx vendor was sufficient to impact their clinical decision. However, they describe a desire for more information to be displayed in the cases where they wish to dive deeper into the reasons behind a potential drug–gene interaction. Meanwhile, some clinicians always read the entire 20 + page PGx report while others consult the whole report only when they are unfamiliar with the PGx vendor or desire more in-depth analysis. All clinicians agreed that PGx reports have an impact on their prescribing behavior: “*the analogy I use with patients is it’s like moving me closer to the dart board. I’m trying to hit the bullseye and I keep missing but lets figure out something that might get you closer*.” (FG1).

#### PGx is rarely a first step

A consensus among the clinicians was that PGx assays were not typically used as a first step during treatment. Although sometimes they served to ease the concerns of the parents, they typically were saved for poor responders or patients with strange symptoms: “*I save it for people who are either getting really bizarre side effects from multiple classes or multiple treatment failures in multiple classes [of medications]*.” (FG1).

#### Trust is important for clinicians to act on PGx reports

An important component of the use of PGx in the clinic is the trust of the source of information by clinicians. Clinicians are more likely to trust the interactions if information of both alleles is provided and if they are familiar with the vendor. Finally, clinicians felt that some vetting by the CDS to ensure that delivered report data are accurate and complete will enhance the use of such assays: “*some vetting up front to make sure what is being pulled and showed to me is OK*.” (FG1).

#### Clinicians regularly interact with and provide PGx reports with patients

In addition to using the PGx reports to impact clinical decisions and treatments, some clinicians also provide physical reports to their patients. These reports improve the communication and discussion of the data as well as serve as a means for patients to keep their PGx data handy for both themselves and for future clinicians: “*I give them a copy of it and say ‘keep this with you at all times especially if you are winding up in an ER, this could be useful information’*.” (FG1).

#### Current shortcomings with the use of PGx at the point of care

Clinicians describe several difficulties in the ability to incorporate PGx reports into the clinical decision and workflow. They wish that PGx reports could be tailored to patient-specific information, such as using CDS to display current and prospective medications of a patient in the common color-coding scheme of reports. Clinicians also struggle with incorporating any PGx report into their clinical workflow if that report is delivered to them in PDF format: “*I pull up genetic testing in our documents so right now its not easy at all*.” (FG3). Finally, they describe the need and desire for PGx data to be up-to-date for their patients and even suggest a discontinuation of using the CDS if the data are not up-to-date.

### Potential negative consequences of CDS in mental health

The use of CDS is just as dependent on its unintended consequences as its intended benefits. The identification and avoidance of potential unintended consequences of CDS prior to integration could potentially increase efficacy and usage by clinicians. The mental health clinicians in this study identified potential major unintended consequences related to alert fatigue, difficulty to input relevant information, the potential increase in the length of time of the patient visit, and their trusts and frustrations in using CDS (Table 3).

#### Alert fatigue

An agreed upon potential unexpected consequence of CDS integration is alert fatigue. While clinicians in this study describe alert fatigue based on unnecessary alerts, they also describe how important alerts, such as lab test reminders, are still necessary and can be helpful to rectify a mistake that has been made. One clinician describes a particularly unnecessary alert that contributes to alert fatigue: “*They give me an interaction alert for a woman when this is a guy I am dealing with*.” (FG1).

#### Difficulty to input relevant information

Another common unintended consequence identified from the focus groups is the inability to input relevant information into the CDS. This information ranged from third-party ratings and scores to hand-written lab reports: “*I wish there was a way to enter data*.” (FG3). Clinicians who consistently use and rely on CDS may be missing pertinent information if not all of the patient data are surfaced: “*A major issue is just pulling data, like a lot of our lab results don’t come into the computer system…it doesn’t really help in centralizing things because they’re not centralized in the first place*.” (FG3).

#### Addition of time to the length of visit

Another potential unintended consequence identified by the focus groups is the potential to increase the length of time of the visit. Clinicians are stretched for time and typically only have 30 min for a follow-up examination. The clinicians prefer the CDS to be at the point of prescribing to avoid having to move through different windows and tabs, and avoid having to go back to a previous step in their workflow, which increases time. Issues with the computational speed of the CDS would also increase the length of time of the visit: *“There were some [widget] speed issues, many things weren’t loading.”* (FG3).

#### Clinicians may lack trust in displayed data

Clinicians described skepticism of the accuracy of described data and information. In many cases, this stemmed from poor past experiences with CDS that failed to provide complete and necessary information to the clinicians. In addition to describing this issue, clinicians suggested that confirmation of the accuracy of the data and vetting on the part of the CDS will ease some of these concerns. “*One of the things that made me nervous, and I think this is just because of what has happened with our EHR here, we’ve had so many situations where things aren’t pulling in correctly and we can’t be 100% sure. So I just found myself, like I’m going to go to [the vendor’s] site because I know that is correct…I just got very nervous, is this correct*?” (FG3).

#### Frustration using the tool

Another negative consequence of CDS incorporation is potential frustration by the clinician when using or trying to use CDS. From usage experience of several types of CDS, clinicians describe a landscape where the ease of use of any tool is important for its incorporation in the clinical workflow. Clinicians were likely to discard CDS that they deemed “supplementary” if there were usage issues. These issues included inconsistencies in performance and display of information, slowness in CDS functions and loading, and even simpler issues such as difficulty opening the CDS: “*There was a lot of effort to get into something that should have been straightforward*.” (FG3).

## Discussion

CDS in mental health has been suggested to improve prescribing behavior and improve communication between clinicians and patients [20]. Similarly, PGx has been shown to increase the efficacy of medications and reduce overall costs within the field of mental health [10–13]. Successful integration of CDS and PGx has been demonstrated in several areas of medicine but so far none has been built with features and desires specific to mental health [17].

Previous studies investigating the integration of PGx and CDS identified several desirable features relating to insurance coverage, CDS workflow integration, and the communication of clinically relevant information [17, 22, 23]. Despite the uniqueness of our study in only including clinicians in mental health, we also describe similarly desirable features, demonstrating that there are some common features related to CDS and PGx that are independent of clinical field. Similar to other studies, alert fatigue was a popular topic among our participating clinicians [22]. The clinicians described the irritation due to unnecessary alerts, and how unnecessary alerts may cause them to miss important alerts. However, the clinicians recognized the usefulness and importance of the presentation of important alerts. This suggests that successful CDS that utilizes alerts must strike a balance for maximum effectiveness and clinician usage.

Henshall et al. [20] recently designed CDS to assist in treatment decision making in psychiatry. Unsurprising because of the similar participant population, the findings in our study are consistent with Henshall et al. in several wide-ranging topics including the use of CDS to improve clinician–patient communication, to better record data, to identify covered medications, and to improve patient compliance. In Henshall et al. [20], the clinicians were concerned about the reliability of data being delivered from a meta-analysis. Similarly, clinicians participating in our study expressed concerns about the reliability of lab reports and PGx data surfaced by CDS. Our study participants stated that they would be more confident in data surfaced in the CDS if it came from reputable vendors and if the CDS vendor vetted it for accuracy and completeness. Clinicians in both studies stated that they are willing to change prescribing decisions based on information delivered through CDS; however, this is dependent on the accuracy and effectiveness of the surfaced data. Therefore, future CDS development should focus on data and information transparency to ease accuracy concerns of clinicians, potentially leading to an increase in usage.

Interestingly, clinicians in Henshall et al. [20] felt that the CDS described in their study could be improved by incorporating biological information to improve personalized medicine. The incorporation of PGx would satisfy this concern, at least in part, and our study describes many features that will increase the effectiveness of future CDS that integrates PGx for use in mental health. Clinicians want these type of data surfaced during prescribing, to have access to full reports when more information than the summary is desired, and to trust the data and the vendor, which is crucial to implement CDS when the prescribing decisions occur in the clinical workflow. Future CDS that successfully meets these requirements will have a better chance of clinician engagement and to improve patient outcomes.

Among the many desired features identified in this study, actionability of the CDS and the performance of the CDS particularly stand out as both are directly related to the specific EHR product. Clinicians describe several features that require actionability of the CDS to the EHR. For example, clinicians would like to write notes through the CDS and store them on the EHR. They would also like to place medication orders through the CDS. Clinicians also describe several points of frustration with CDS performance, many of which are directly related to the EHR. Clinicians experience slowness in performance of CDS including issues with tasks as simple as opening the tools. They also describe how they lack trust in the information displayed in CDS. Even in instances where all of the information within the EHR is successfully surfaced by the CDS, they know that the EHR is incomplete; therefore, the clinicians fail to trust much of the information displayed by CDS. These frustrations are particularly noteworthy because in our study, clinicians specifically stated that similar experiences have lead them to abandon associated CDS completely.

Because CDS actionability and the computational performance of CDS also depend upon EHR features and performance, any venture to produce CDS must also take into account the integration with each specific EHR. Unfortunately, many EHRs have limitations in that the flow of information is incapable of writing data from the CDS to the EHR, demonstrating that inherent limitations of some EHRs also limit the ability and full effectiveness of third-party CDS. This EHR limitation directly impairs many of the features desired by clinicians, including the ordering of medications and the delivery of notes from the CDS to the EHR. Another EHR flaw that impacts CDS effectiveness is the incomplete record within the EHR that many CDS tools rely on to pull from and display that information. In our study, clinicians lamented the incompleteness of labs and even stated that they do not trust the lab results in their own EHR and instead go directly to lab vendor websites when they view that information. These EHR limitations severely reduce the performance of CDS and this must be taken into account before any development of CDS.

This study also identifies a desire to have prospective versus retrospective actionability by the clinician, both related to PGx interactions and to medication coverage information. Clinicians described the desire to overlay PGx data when choosing to prescribe medications. One solution suggested by the clinicians is to color code the medications at the point of prescribing based on the predicted PGx interactions. Additionally, this and past studies have shown that clinicians express a desire to know what medications are covered by their patient’s health insurance plan prior to prescribing [20]. These two features will allow clinicians to preemptively use PGx and cost data to assist them in the selection of medications for their patients. This prospective decision making has the ability to increase efficacy and reduce healthcare costs [24].

We have identified several limitations of our study. The first limitation is related to the participant characteristics in that all of the participating clinicians practice medicine in the urban setting. Urban clinicians may not have similar desired features compared to clinicians practicing in more rural areas. Patients in rural areas have limited access to specialists, increasing the likelihood they will be seen by general practitioners [25]. This has broad implications in the field of mental health as patients exceeding a minimally adequate treatment by general physicians are only 12.7% compared to 48.3% when seen by mental health specialists [26]. Therefore, the recommendations in this study may be more applicable to specialists in the urban setting versus other areas.

Another important limitation of this study is that all of the participating clinicians primarily use the same EHR system. As noted, many features and limitations of CDS are inherently tied to the EHR, such as CDS actionability, display of relevant information, CDS performance, and workflow. The EHR these clinicians use may have biased them toward their response to which features would be most beneficial. Sampling from clinicians using different EHR systems may provide broader and more generalized feedback on desired CDS and PGx features by mental health clinicians.

## Conclusions

This study used focus groups with mental health clinicians and a prototype CDS software solution to identify desired features related to the integration of CDS and PGx into the EHR in a mental healthcare setting. Successful integration and delivery of these desired features may lead to improved patient outcomes and increase the clinician usage of the CDS. The results from this study will assist with the design and construction of future mental health CDS with integrated PGx to maximize the CDS effectiveness.

## Data Availability

All data generated or analyzed during this study are included in this published article.

## Abbreviations

CDS: clinical decision support
EHR: electronic health record
PGx: pharmacogenomics
FG: focus group
PHQ9: Patient Health Questionnaire
BMI: body mass index
BP: blood pressure
FDA: Food and Drug Administration

## Footnotes

1 https://www.nimh.nih.gov/health/statistics/mental-illness.shtml.

2 https://www.fda.gov/medical-devices/safety-communications/fda-warns-against-use-many-genetic-tests-unapproved-claims-predict-patient-response-specific .
